# Sulfur deficiency-induced genes affect seed protein accumulation and composition under sulfate deprivation

**DOI:** 10.1093/plphys/kiab386

**Published:** 2021-08-10

**Authors:** Fayezeh Aarabi, Apidet Rakpenthai, Rouhollah Barahimipour, Michal Gorka, Saleh Alseekh, Youjun Zhang, Mohamed A Salem, Franziska Brückner, Nooshin Omranian, Mutsumi Watanabe, Zoran Nikoloski, Patrick Giavalisco, Takayuki Tohge, Alexander Graf, Alisdair R Fernie, Rainer Hoefgen

**Affiliations:** 1 Max-Planck-Institute of Molecular Plant Physiology, Am Mühlenberg 1, 14476 Potsdam-Golm, Germany; 2 Center of Plant Systems Biology and Biotechnology, 4000 Plovdiv, Bulgaria; 3 Department of Pharmacognosy, Faculty of Pharmacy, Menoufia University, Gamal Abd El Nasr St, Shibin Elkom, Menoufia 32511, Egypt; 4 Graduate School of Biological Sciences, Nara Institute of Science and Technology, Ikoma, Nara 630-0192, Japan; 5 Bioinformatics, Institute of Biochemistry and Biology, University of Potsdam, Karl Liebknecht Str. 24-25, 14476 Potsdam-Golm, Germany; 6 Max Planck Institute for Biology of Ageing, Joseph Stelzmann Str. 9b, Cologne 50931, Germany

## Abstract

Sulfur deficiency-induced proteins SDI1 and SDI2 play a fundamental role in sulfur homeostasis under sulfate-deprived conditions (−S) by downregulating glucosinolates. Here, we identified that besides glucosinolate regulation under –S, SDI1 downregulates another sulfur pool, the S-rich 2S seed storage proteins in Arabidopsis (*Arabidopsis thaliana*) seeds. We identified that MYB28 directly regulates 2S seed storage proteins by binding to the *At2S*4 promoter. We also showed that SDI1 downregulates 2S seed storage proteins by forming a ternary protein complex with MYB28 and MYC2, another transcription factor involved in the regulation of seed storage proteins. These findings have significant implications for the understanding of plant responses to sulfur deficiency.

## Teaser

Sulfur Deficiency Induced1 (SDI1) upregulates S-poor seed storage proteins in favor of S-rich seed storage proteins.

## Introduction

Seed storage proteins (SSPs) are considered as an essential source of nitrogen, carbon, and sulfur during seed germination, and their amount varies relative to the availability of nutrients in the soil (Higashi et al., 2006). In Arabidopsis seeds, two major types of storage proteins exist, 12S globulins or cruciferins (saline-soluble) and 2S albumins or arabidins (water-soluble; Shewry et al., 1995). SSPs of different plants show a common behavior in response to sulfur deficiency (−S). Sulfur-rich proteins, for instance, 12S globulins and 2S albumins of Arabidopsis or 11S globulins (glycinin) of soybean, are decreased (Hirai et al., 1995; Higashi et al., 2006), and sulfur-poor SSPs, such as β-conglycinin (the 7S globulin) of soybean, accumulate (Hirai et al., 1995). By this mechanism, plants can maintain nitrogen sources for their growth in the form of seed proteins even under sulfur-deficient conditions (Higashi et al., 2006). Thus far, very few studies have investigated the regulation of SSPs in response to −S, and the underlying molecular mechanism involved in triggering the differential protein composition in seeds is poorly understood. Regulation of SSPs has been reported to occur at the transcriptional and post-translational levels under −S. It has been reported that the gene encoding the β subunit of β-conglycinin is upregulated at the transcriptional level (Hirai et al., 1995, 2003; Kim et al., 1999). Meanwhile, application of *O*-acetylserine (OAS), the immediate precursor of cysteine synthesis, to immature soybean cotyledons resulted in a similar pattern of SSP accumulation to that seen under sulfur deficiency (Kim et al., 1999; Hirai et al., 2003). Therefore, OAS is considered a regulator of SSP gene expression (Hirai et al., 2003). *Sulfur deficiency induced* (*SDI*) genes, *SDI1 and SDI2*, have been long identified as OAS-responsive genes (Hubberten et al., 2012; Aarabi et al., 2015), and have key roles in the downregulation of the S-rich secondary metabolites, glucosinolates (GSLs), in shoots and roots of Arabidopsis via interaction with MYB28 in the nucleus (Aarabi et al., 2016).

Here, we demonstrate that *SDI1*, which is also highly expressed in seeds under sulfur deficiency (−S), has an additional role in modulating the SSP profile in favor of S-poor proteins via interaction with MYC2 transcription factors (TFs), known to participate in this process (Gao et al., 2016), and MYB28, demonstrated here. Therefore, in seeds, SDI1 coordinately downregulates the two main sulfur-rich pools of the sulfur assimilation pathway and metabolism under low sulfate conditions: GSLs and S-rich SSPs. Metabolome data also reveal distinct metabolic changes in seeds upon *SDI* perturbation including amino acids, organic acids, and sugars, mimicking the responses seen under −S, validating further SDI-specific roles under S deprivation (Nikiforova et al., 2005; Bonnot et al., 2020). Besides, profiles of the secondary metabolites of *SDI* transgenic seeds, revealed substantial changes in sinapate esters, with some known to possess antinutritive properties in *Brassica napus* seeds for animal feed and human nutrition (Milkowski and Strack, 2010). Results of this study shed light on deciphering the molecular mechanism stimulating the alteration in seed protein and metabolome composition and may be utilized in future studies to improve grain nutritional properties in crop plants.

## Results

### *SDI* expression in Arabidopsis seeds

**Figure 1:**
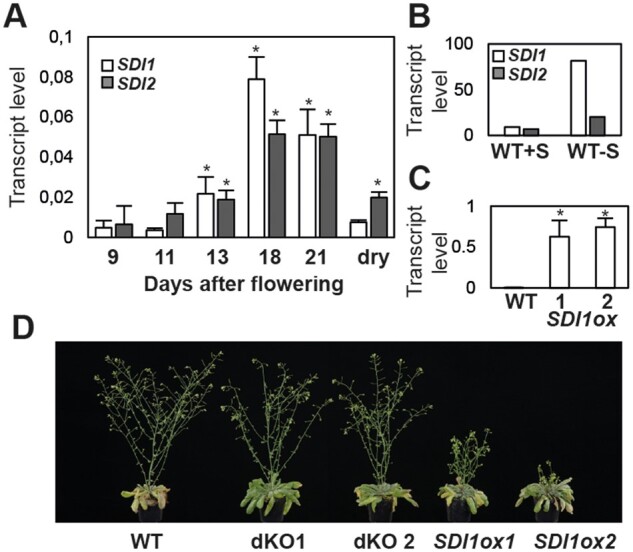
*SDI* expression in Arabidopsis seeds and morphological phenotype of the transgenic lines. A, Relative expression of *SDI1 and SDI2* in Arabidopsis seed throughout different seed developmental stages, assayed by qRT-PCR. Asterisks demonstrate significant changes versus the values at 9 DAF (*t* test; *P* < 0.05). Three biological replicates and two technical replicates were used at 9 and 13 DAF, and two biological replicates, and two technical replicates were used at 11, 18, 21 DAF, and dry seed stages. B, Transcript levels of *SDI1 and SDI2* in WT Arabidopsis seeds, grown under +S (1.5-mM sulfate) and –S (30-µm sulfate) conditions. The graph is depicted based on the microarray data from Higashi et al. (2006), based on one biological replicate. C, Transcript levels of *SDI1* in Arabidopsis seeds of WT and *SDI1ox* lines quantified at 11 DAF. D, Growth phenotype of the *SDI* transgenic lines grown under long-day conditions for 6 weeks. Error bars indicate standard deviation in (A) and (C).

To analyze SDI function and monitor its expression in seeds, we extracted RNA from wild-type (WT) seeds at six stages of development: 9 d after flowering (DAF), representing the late cotyledon (COT) stage, 11 and 13 DAF, representing the mature green (MG) stage, 18 DAF, representing the post MG (PMG) stage, and 21 DAF and dry seeds, representing the desiccation period. Reverse transcription quantitative polymerase chain reaction (RT-qPCR) analysis of the expression of *SDI* genes showed that *SDI1 and SDI2* transcripts increased linearly during seed maturation, reached maximum expression levels at 18 DAF, and began to decrease after the onset of seed desiccation (Figure 1A). Similarly, previously published microarray data demonstrated a peak of transcript accumulation of *SDI1* at the onset of seed maturation, more specifically, at peripheral (PEN), chalazal (CZE), and micropylar (MCE) subregions of the endosperm (Supplemental Figure S1; Belmonte et al., 2013). Previous studies showed that −S induces the expression of *SDI1 and SDI2* in developing seeds of Arabidopsis (Higashi et al., 2006). *SDI1* responds to a greater extent (i.e. 80-fold increase) to sulfur starvation than *SDI2* (i.e. 20-fold increase; Figure 1B). These results suggest that SDI proteins might have a function at the late stage of seed development and that under −S *SDIs* are induced (Higashi et al., 2006) independent of the developmental control. The above-mentioned expression data, both at normal seed developmental stage and at −S demonstrated higher expression levels of *SDI1* than *SDI2* (Figure 1), and therefore we aimed to study more specifically the role of SDI1 on seed metabolism and development. To do so, we utilized the *sdi1sdi2* double knockouts (dKOs), and transgenic *SDI1* overexpressing (ox) lines generated under the control of the constitutive CaMV 35S promoter. Overexpression of *SDI1* in developing seeds of ox lines was validated by RT-qPCR (Figure 1C). *SDI1ox1 and ox2* lines showed 173.3- and 206.2-fold increase in the expression of *SDI1*, respectively, at 11 DAF in developing seeds compared to WT (Figure 1C). Six-week-old *SDI1ox* plants grown under short-day or long-day conditions exhibited shorter inflorescence stems than those of WTs, while dKOs did not display any growth phenotypes (Figure 1D).

### Transcript profiling of developing *SDI* transgenic seeds indicates a role for SDI in modulating genes encoding SSPs

**Figure 2:**
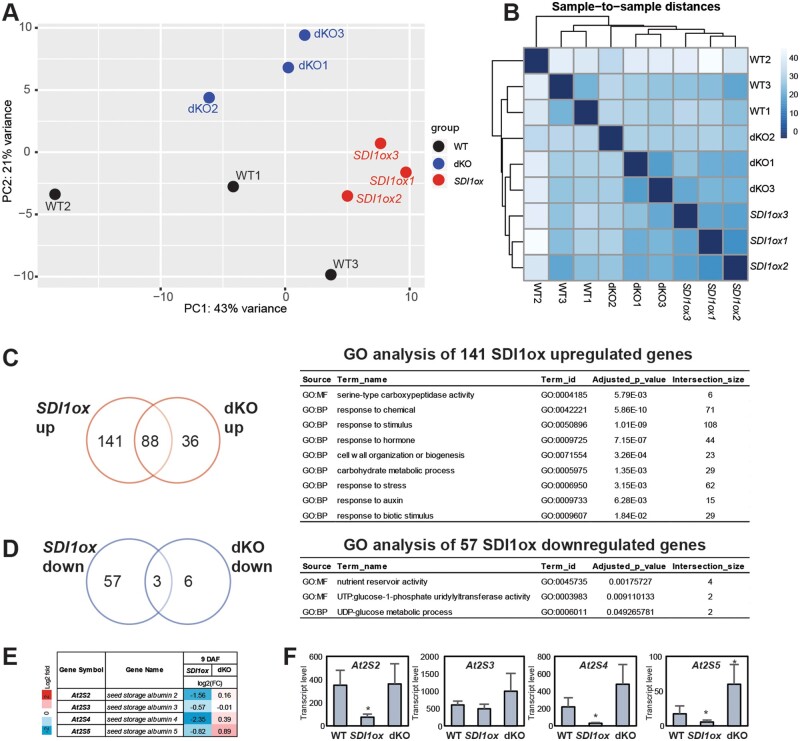
Differential effects of *SDI1* overexpression and *sdi1sdi2* knockout on the seed transcriptome at 9 DAF. A, PCA plot explains the variance in normalized read counts from three biological sample libraries of, *SDI1ox*, dKO, and WT at 9 DAF along PC1 or *X*-axis and PC2 or *Y*-axis. B, Hierarchical clustering of the heatmap of the sample-to-sample distances demonstrates similarities and dissimilarities between samples. Venn diagrams of the significantly up- and downregulated genes in seeds of ox and dKO lines versus WTs depicted in red (C) and blue (D), respectively (selection of 1.5-fold threshold, FDR < 0.05) and the GO terms enriched for the *SDI1ox*-specific up/downregulated genes are depicted. Transcript profile of the genes encoding 2S-SSPs, is depicted in (E) and (F) quantified by RNA-seq and qRT-PCR, respectively. Data in F are mean ± sd of six replicates (three biological, two technical). Asterisks demonstrate significant changes, *t* test, *P* < 0.05. Error bars indicate standard deviation.

To assess whether *SDI* overexpression or knockout has any effect on global transcription, the transcriptomes of developing seeds of *SDI* transgenic lines were determined by RNA-seq. Libraries have been generated from total RNAs extracted from green developing seeds of the WT, *SDI1ox*, and dKO lines at 9 DAF. We examined differentially expressed genes (DEGs) between genotypes by DESeq2. Although the biological replicates showed association and clustered together in the principal component analysis (PCA), genotypes did not show extremely clear separations (Figure 2A). Similarly, sample heatmaps demonstrated high similarities between the genotypes, despite the biological replicates of each genotype being clustered together (Figure 2B). This resulted in only a small number of differentially expressed genes between the transgenic lines and the WTs as reflected in the corresponding MA plots (Supplemental Figure S2). Among the 229 significantly upregulated genes in *SDI1ox* line compared to WT (above 1.5-fold, false discovery rate [FDR] < 0.05), 141 genes were differentially expressed in comparison to dKO, which revealed significantgene enrichment (GO) for responses to abiotic and biotic stimulus, hormone, cell wall organization and biogenesis, and serine-type carboxypeptidase activity (Figure 2C). Several genes encoding serine carboxypeptidase-like (SCPL) enzymes were among the significantly upregulated genes in *SDI1ox* seeds, including *SCPL8*, *10*, *11*, *13*, *34*, and *51* (Supplemental Data Set 1). Among these genes, *SCPL8* (also annotated as *SINAPOYLGLUCOSE 1*, *SNG1*; At2g22990) was found to be responsible for encoding the sinapoyl-Glc:malate sinapoyltransferase (SMT) enzyme which catalyzes the conversion of sinapoyl-Glc to sinapoyl-malate (SinM) (Fraser et al., 2007). SMT has been also shown to have a function in catalyzing the formation of 1,2-disinapoyl-Glc along with *SCPL13* (At2g22980; Fraser et al., 2007). *SCPL10* (At2g23000) was reported to encode sinapoyl-Glc:anthocyanin sinapoyl transferase, which is an enzyme that synthesizes sinapoylated anthocyanins in Arabidopsis (Fraser et al., 2007). These data suggest a specific function for SDI1 in sinapate ester metabolism in Arabidopsis seeds.

GO enrichment of the 57 *SDI1*-specific downregulated genes demonstrated enrichments for nutrient reservoir activity, including those responsible for 2S SSP synthesis, *At2S2* (*SESA2*), *At2S4* (*SESA4)*, and *At2S5* (*SESA5*), and UTP:glucose-1-phosphate uridylyltransferase activity, including those responsible for uridine diphosphate (UDP)-glucose metabolic process, *UGP1*, and *UGP3* (Figure 2D;Supplemental Data Set 1). Among the *2S* genes, *At2S4* exhibited the most prominently decreased expression (approximately five-fold), and *At2S2* and *At2S5* transcripts were decreased about three- and two-fold in *SDI1ox* seeds, respectively (Figure 2E;Supplemental Table S1). *At2S5* expression was also significantly upregulated in dKO seeds versus WT (Figure 2E;Supplemental Table S1).

Together, transcript data indicate that *SDI* perturbation in seeds modulates the expression of some genes involved in specific metabolic pathways including sinapate esters deriving from shikimate/phenylpropanoid pathway and 2S-rich SSPs.

To validate the observed DEGs involved in SSP synthesis, we quantified the expression levels of selected genes involved in seed protein accumulation by RT-qPCR, including *At2S1*-*At2S5*, and *At12S1*-*At12S4*, encoding the 2S and 12S SSPs, respectively, and those encoding the key TFs involved in seed maturation and regulation of the SSP synthesis, including *LEC1*, *LEC2*, *ABI3*, *FUS3*, and *bZIP25* (Supplemental Table S2). RT-qPCR demonstrated a high association with the RNA-seq data (Supplemental Table S2 and Supplemental Figure S3), and genes encoding 2S proteins, *At2S2*, *At2S4*, and *At2S5* were significantly downregulated in *SDI1ox* and no significant changes could be observed in the expression of other genes responsible for regulation or synthesis of SSPs (Figure 2F;Supplemental Table S2).

**Figure 3:**
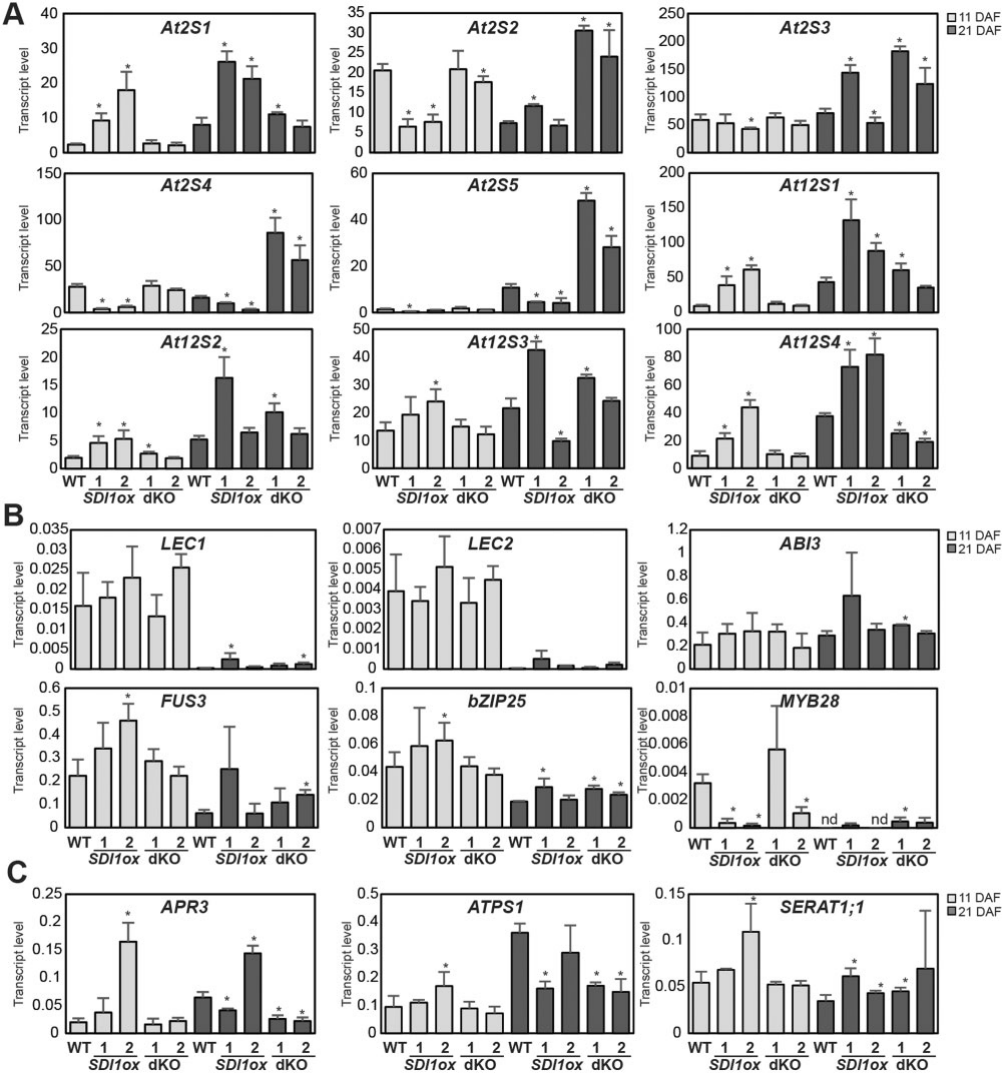
Expression levels of seed protein and S assimilation-related genes at 11 and 21 DAF. Relative expression of genes encoding 2S, and 12S proteins (A), TF regulating the expression of SSPs (B), and a selection of genes involved in sulfate assimilation and Cys synthesis (C) at two developmental stages, 11 and 21 DAF is assayed by qRT-PCR in seeds of the WT, and the transgenic lines. Data are mean ± sd of four replicates (two biological, two technical). Asterisks demonstrate significant changes, *t* test, *P* < 0.05.

To further validate the observed transcript phenotypes, we incorporated two other time-points of seed development for transcript analyses, including 11 DAF and 21 DAF, and two independent lines for each genotype were investigated. Similar to the observed phenotype at 9 DAF, RT-qPCR revealed that among the 2S encoding genes, *At2S4*, and *At2S5* were significantly downregulated in *SDI1ox* lines at both 11, and 21 DAF, and *At2S2* was significantly downregulated at 11 DAF. In contrast, dKO lines showed a significant upregulation of *At2S2*, *At2S3, At2S4*, and *At2S5* expression levels (Figure 3A;Supplemental Table S3). Among these genes, *At2S3* displayed the least alteration, and *At2S4 and At2S5* were affected the most (Figure 3A;Supplemental Table S3). Interestingly, some of the genes encoding SSPs were differentially regulated compared to that seen above; contrary to the reduced expression of *At2S2*, *3*, *4*, and *5* in *SDI1ox* lines, *At2S1 and At12S1*, *12S2*, and *12S4* were significantly upregulated in *SDI1ox* lines at 11 and 21 DAF, accompanied by the downregulation of *At12S4* in dKO lines at 21 DAF (Figure 3A;Supplemental Table S3). Furthermore, none of the TFs regulating SSP encoding genes were differentially regulated between the *SDI1ox* and dKO lines, for example, *LEC1* expression level was significantly increased ∼10-, and ∼5-fold in both *SDI1ox1* and dKO2 lines, respectively at 21 DAF (Figure 3B;Supplemental Table S3). A similar expression pattern was observed for *bZIP25* (Figure 3B;Supplemental Table S3). This observation indicates a complex relationship between the *SDI* genes and the TFs regulating the SSPs. However, investigating the expression patterns of *SDI* genes and the TFs regulating the SSPs over different developmental stages in WT seeds demonstrated an antagonistic relationship between them (Supplemental Figure S4).

As *SDI* genes are −S marker genes (Aarabi et al., 2016), expression levels of some of the genes involved in sulfur assimilation and cysteine biosynthesis, such as *ATPS1*, *APR3*, and *SERAT1;1* were examined, in order to verify whether the transcriptome of the sulfur assimilation pathway was altered. *SDI1ox2* line showed ∼8.5-, ∼1.7-, and ∼2-fold increase in the expression of *APR3*, *ATPS1*, and *SERAT1;1* at 11 DAF, respectively, and dKO lines showed significant downregulation of *APR3* and *ATPS1* expression levels at 21 DAF (Figure 3C). APR3 was also significantly upregulated in *SDI1ox2* at 21 DAF (Figure 3C). The upregulation of *APR3 and ATPS1* in *SDI1ox* lines indicated a response resembling a sulfur limitation response, especially in the *SDI1ox2* line.

### Effects of SDI1 perturbation on the seed metabolome

**Figure 4:**
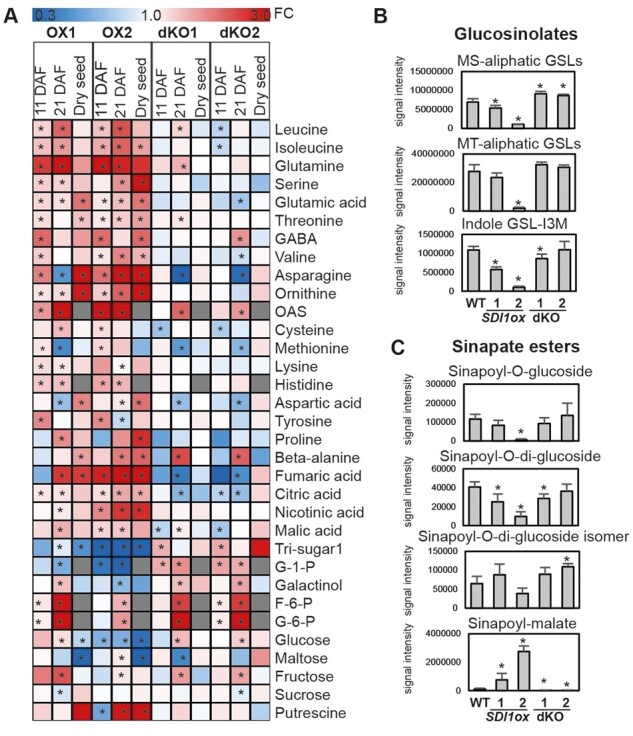
Metabolite profiles of developing and dry seeds of the *SDI* transgenic lines. A, Heatmap shows fold change ratios of primary metabolites in seeds of the *SDI* transgenic lines compared to WTs quantified in developing seeds at 11 and 21 DAF and in dry seeds. Statistically significant differences (*t* test; *P* ≤ 0.05, *n* = 6) between the WT and the transgenic lines are shown by asterisks on the heatmap. GSL contents (B) and sinapate esters (C) in dry seeds of WTs and the *SDI* transgenic lines, quantified by LC-MS. In B and C bars and error bars show means and sd of four replicates. **P < 0.01, *P < 0.05, indicate significant differences detected using *t* test between WT and the transgenic lines.

To evaluate SDI effects on the seed primary metabolome we performed high-throughput gas chromatography–mass spectroscopy (MS) analysis on developing and dry seeds at 11 and 21 DAF. Metabolomics revealed major changes in the levels of amino acids, sugars, and organic acids at both developing and dry seed stages (Figure 4A). We were able to quantify OAS levels in seeds only at 11 and 21 DAF, demonstrating that *SDI1ox* lines accumulated up to approximately four- to five-fold OAS levels (Figure 4A). OAS also accumulated in dKO lines at 21 DAF but to a lesser degree than compared with the ox lines (Figure 4A). dKO lines also demonstrated decreased Cys and Met levels at 11, and 21 DAF, respectively. In contrast, ox lines showed a slight increase in Met levels at 11 DAF (Figure 4A). In dry seeds, the Cys-derived amino acid, cystathionine, which is an intermediate in Met synthesis, increased strongly in *SDI1ox2* by ∼18-fold, whereas Cys and Met levels remained almost unaffected (Supplementary datasets 2). The data demonstrated that many other free amino acids were significantly accumulated in ox lines, among them glutamic acid (Glu), asparagine (Asn), and ornithine in both ox lines at all three developmental stages (Figure 4A). Glutamine (Gln), leucine (Leu), isoleucine (Ileu), and histidine significantly accumulated in both ox lines at 11 and 21 DAF (Figure 4A). In contrast to the ox lines, dKO lines did not show dramatic changes in the level of amino acids, and only aspartic acid (Asp) levels were slightly and Asn levels strongly decreased at 21 DAF (Figure 4A). Furthermore, expression levels of the genes involved in nitrate assimilation, *NITRATE REDUCTASE1* and *2* (*NIA1 and NIA2*) in the *SDI1ox* line at 9 DAF were also increased (Mohn et al., 2019; Supplemental Data Set 1). This might indicate a higher reduction of nitrate into ammonium, thereby incorporating more nitrogen into amino acids. Furthermore, ion measurements revealed a significant accumulation of nitrate in dry seeds of *SDI1ox* lines, whereas sulfate remained unaffected (Supplemental Data Set 2 and Supplemental Figure S5).

*SDI1ox* lines also demonstrated a tendency of increase in the level of organic acids, among them the content of fumaric acid increased by ∼2.5-fold in *SDI1ox1* at 21 DAF, and strongly accumulated in *SDI1ox2* in all developmental stages (Figure 4A). Nearly similar behavior could be observed for nicotinic acid, malic acid, and citric acid (Figure 4A). Conversely, dKO lines at 21 DAF showed significantly decreased levels of fumaric and citric acid (Figure 4A). In contrast to amino acids and organic acids, sugars displayed a general reduction in dry seeds of ox lines. Except for the unchanged levels of sucrose and fructose, the levels of glucose, 1,6-Anhydro-glucose, maltose, and gentiobiose were markedly reduced in dry seeds of ox lines (Supplemental Data Set 2). Similarly, glucose was significantly reduced in ox2 at 11 and 21 DAF, and accumulated in dKO lines at 21 DAF (Figure 4A). Glucose 1-phosphate was also markedly decreased in ox lines but accumulated in dKO lines at 11 and 21 DAF (Figure 4A).

Moreover, to evaluate SDI effects on the metabolome of secondary compounds in Arabidopsis seeds, we performed high-throughput liquid chromatography with tandem mass spectrometry (Tohge and Fernie, 2010; Salem et al., 2016, 2017) analyses on dry seeds of the *SDI* transgenic lines. We observed similar metabolic changes in the levels of GSLs to those seen in leaves and roots of the *SDI* transgenic lines (Aarabi et al., 2016). Most of the methylsulfinylalkyl-GSLs, and some of the methylthioalkyl-GSLs accumulated significantly in dKO1 and dKO2 and were reduced in *SDI1*ox lines (Figure 4B;Supplemental Data Set 2). Indolyl-3-methyl GSL was also significantly reduced in *SDI1*ox lines (Figure 4B). Besides the observed alterations in GSL levels, SDI perturbation affected other secondary metabolites in seeds, including some of the flavonols and sinapate esters (Figure 4C;Supplemental Data Set 2). Both *SDI1ox* lines displayed increased levels of quercetin-3-O-(2"-O-rhamnosyl)-glucoside-7-O-rhamnoside (Q3GR7R; Supplemental Data Set 2). Among sinapate esters, in contrast to the severe reduction of two sinapoyl-O-glucoside (SinG1, sinapoyl-) and sinapoyl-O-di-glucoside (SinGG) in *SDI1ox* lines, SinM was increased strongly by 6.5- to 23-fold (Figure 4C). In contrast, dKO lines showed a severe reduction of SinM (Figure 4C).The strong overaccumulation of SinM at the expense of sinapoyl-glucosides in dry seeds of the ox lines is in line with the observed upregulation of *SNG1* expression level, encoding the enzyme responsible for conversion of sinapoyl-Glc to SinM. Furthermore, total protein contents and the lipid profile of the dry seeds did not show significant changes between the transgenic lines and controls (Supplemental Figure S5). These data show that *SDI* perturbation has a significant impact on the primary and secondary metabolome of the Arabidopsis seeds but not on protein amounts and lipid composition.

### SDI represses accumulation of 2S SSPs

**Figure 5:**
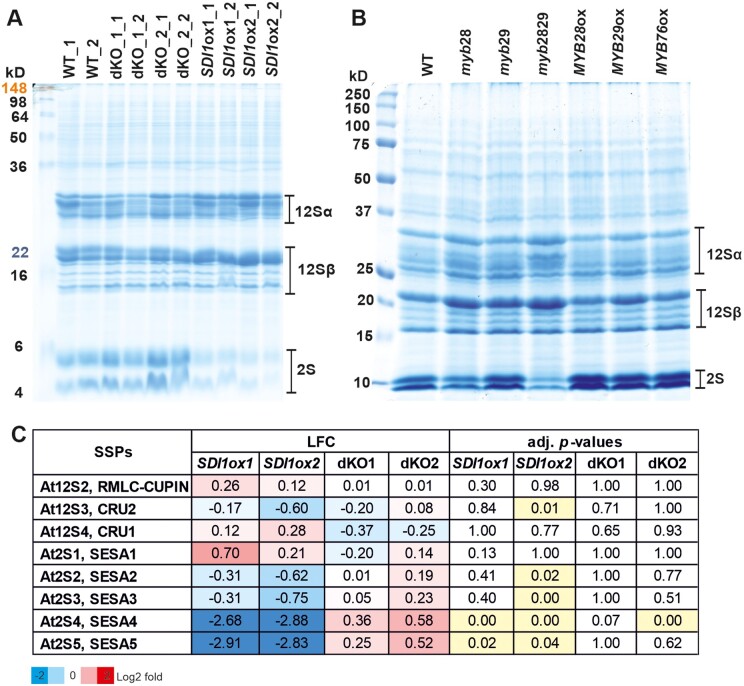
Seed protein analysis of *SDI and MYB* transgenic lines. A, Protein profiles of dry seeds of the *SDI* transgenic lines. Protein extracts were prepared from two biological and two technical replicates for each transgenic line. Total protein extracts from the dry mature seeds were prepared as described in the “Material and methods” and proteins were separated by SDS–PAGE. α- and β-subunits of 12S cruciferins and 2S albumins are indicated with lines on right side of the gel. B, Protein profiles of dry seeds of the *MYB* transgenic lines. Protein extracts were prepared and SDS–PAGE was performed as described in a SeeBlue Plus2 Pre-stained Protein Standard (Life technologies) was used as a molecular weight standard in A, and B. C, Profile of SSPs in dry seeds of the *SDI* transgenic lines, quantified by MS. Values indicate log2 fold ratios (LFC) of normalized protein abundances between *SDI* transgenic lines and the WT. The statistical analysis was done with Benjamini and Hochberg multiple testing adjustment and significant changes (P-adj ≤ 0.05, four biological replicates) are highlighted in yellow in the table.

Considering the fact that −S affects the protein profile of seed protein reserves (Naito et al., 1994; Hirai et al., 1995) and that *SDI* is highly induced in developing Arabidopsis seeds upon S stress (Higashi et al., 2006), we aimed at investigating the impact of ectopic expression of *SDI and SDI* gene knockout on the protein profile of mature Arabidopsis seeds. Sodium dodecyl sulphate–polyacrylamide gel electrophoresis (SDS–PAGE) analysis showed that seeds of dKO lines contained a slightly increased amount of 2S albumins (a sulfur-rich protein), and conversely, *SDI1ox* lines contained remarkably reduced levels of 2S albumins compared to the WT (Figure 5A). Density quantification of the protein bands demonstrated that *SDI1ox* lines contained about 30% of WT 2S albumin contents (Supplemental Figure S6). A strong trend toward accumulation of 12S and 2S proteins could be also observed in *SDI1ox* and dKO lines, respectively (Supplemental Figure S6). To precisely quantify the amount of SSPs in transgenic lines and verify the observed phenotype, we next performed mass spectrometry (MS) analysis of protein extracts of mature seeds. The results obtained from MS were in accordance with the SDS–PAGE results especially with respect to *SDI1*ox line, as two isoforms of 2S albumins, SESA4 and SESA5 were strongly downregulated in both *SDI1*ox lines (Figure 5C). Levels of SESA4 and SESA5 in seeds of ox lines comprised only about 13%–15% and 13%–14% of the WT levels, respectively. Though a trend for an increase of these peptide levels could be seen in dKO lines, the increase is statistically significant only for the SESA4 in line dKO2 (Figure 5C). Furthermore, the levels of SESA2 and SESA3 were significantly lowered in *SDI1ox*2 relative to the WT (Figure 5C). Moreover, β subunits of 12S globulins appeared to be increased in *SDI1*ox lines from the SDS–PAGE analysis, which was also reflected in the expression data (Figure 3A). However, MS analysis did not show significant changes in the levels of 12S proteins detected, including, At12S2 and At12S4 proteins (Figure 5C). At12S3 appeared to be significantly reduced in *SDI1*ox2 line which was in line with the reduced expression level of *At12S3* at 21 DAF (Figures 3A and 5C). In general, protein and transcript profiles of SSPs were positively associated.

### MYB28, MYB29, and MYB76 function in seed protein accumulation

**Figure 6:**
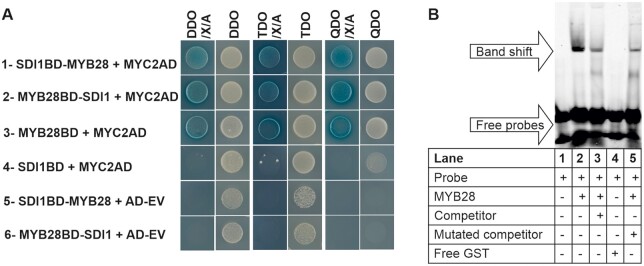
Investigation of ternary protein complex formation among SDI1, MYB28, and MYC2 and binding activity of MYB28 to SESA4 promoter. A, Panels 1 and 2 demonstrate the positive interaction among SDI1, MYB28, and MYC2 in Y3H screenings. In panel 1 SDI1BD-MYB28 expressed SDI1 fused to the DNA-BD (Gal4 DNA-binding domain) as well as MYB28 expressing as the bridging protein. In panel 2, MYB28BD-SDI expressed MYB28 fused to the DNA-BD domain and SDI1 as the bridging protein. MYC2AD was used as prey expressing MYC2 fused to the activation domain (AD). Cotransformations of prey empty vector (AD-EV) with pBridge baits were performed as negative controls in the last two panels. Y2H assays were performed as in panels 3 and 4 by co-transformation of the respective prey and bait constructs grown on dropout plates with or without X-α-Gal (X) and Aureobasidin (A). The double (DDO), triple (TDO), and quadruple (QDO) dropout media are described in the “Material and methods” section. B, EMSA shows the binding activity of MYB28 to the promoter of *At2S4* or *SESA4*. The probe sequences were designed as described in the “Material and methods”. The presence or absence of the reagents in each lane is indicated with (+) and (−), respectively. Adding the unlabeled competitor in molar excess reduced the signal intensity. However, we could not confirm the specificity of the interaction between the selected MYB cis-element in the promoter of SESA4 and MYB28 because adding the mutated version of the unlabeled probe diminished the binding intensity.

SDS–PAGE analysis on protein extracts of *MYB* transgenic lines showed that 2S proteins accumulation behaved in a manner opposite to that of the *SDI* transgenic lines as *MYB28*, *MYB29*, and *MYB76* OX lines contained remarkably higher levels of 2S albumins, and inversely, *myb28 and myb2829* knockout lines contained reduced levels of 2S albumins compared to the WT (Figure 5B). SDS–PAGE also showed a trend for an increase of the levels of β subunits of 12S globulins in *myb* knockout lines. From these data, we can conclude that MYB28, MYB29, and MYB76 can have redundant functions in regulating the seed protein accumulation in Arabidopsis seeds and, thus, we hypothesize that the suppression of SSPs via SDI might be caused by its inhibitory function on the MYB28 transcription factor as we identified in previous studies an inhibitory SDI interaction with MYB28 (Aarabi et al., 2016). The inhibitory effect of SDI on MYB28 had been also reflected in the transcript data, as *SDI1ox* lines showed a strong reduction in *MYB28* transcripts at 11 DAF, and dKO1 showed increased expression of *MYB28* at 21DAF; however, it was barely detectable in other genotypes at that developmental stage (Figure 3B). It is worth mentioning that the interaction of SDI1 with all three MYB TFs has been confirmed by Y2H analysis in our previous studies (Aarabi et al., 2016). Furthermore, recent studies identified that MYC2, MYC3, and MYC4 positively regulate Arabidopsis SSP accumulation (Gao et al., 2016). *myc234* triple mutants contained a reduced amount of 2S albumins compared to the WT similar to that of *myb28myb29* dKOs (Gao et al., 2016). Furthermore, Arabidopsis MYC2, MYC3, and MYC4 are additional regulators of GSL biosynthesis via a direct interaction (through JID domain) with MYB TFs and, hence, regulating aliphatic GSLs through positive interaction with MYB28, MYB29, and MYB76 (Schweizer et al., 2013). Therefore, we hypothesize that MYC and MYB TFs might function synergistically in seeds to control SSPs and that SDIs might interact in a ternary protein complex with MYC and MYB TFs to confer its inhibitory effect. To test this hypothesis, we took advantage of the Y3H approach using the pBridge vector which allowed investigating ternary protein complex formation by SDI1, MYB28, and MYC2. First, we confirmed again the physical interaction between MYB28 and MYC2 in an Y2H system (Figure 6A, row 3). Then, when we used SDI1BD as bait and MYC2AD as prey, we could see that SDI1 does not directly interact with MYC2 (Figure 6A, row 4). However, when we used MYB28 or SDI1 as the bridging proteins in Y3H combinations we could see the activation of the reporter in both cases (Figure 6A, rows 1 and 2), which allows the conclusion, that SDI1 forms a ternary protein complex with MYB28 and MYC2. Additionally, we conclude that MYB28 acts as a “bridge”, interacting with SDI1 and MYC2 that do not directly interact with each other, and SDI1 does not interfere with MYB28-MYC2 interaction. Next, to prove the role of MYBs in the regulation of genes encoding SSPs, we performed electrophoretic mobility shift assays (EMSAs) using MYB28 protein and fluorescently labeled (5′-DY-682) and unlabeled DNA probes (competitor) to the promoter sequence of *At2S4* (Figure 6B;Supplemental Figure S7). To design the probes, we performed a motif enrichment analysis in all *SESA* promoters using MEME program (Bailey et al., 2009). Out of the three enriched motifs, we selected motif 2 located in the promoter of *SESA4* for generating the probe as it harbored a putative MYB R2R3 binding site (MybBS; Schweizer et al., 2013) and neighbors a MYC2 core binding site, known as G-box (CACGTG), and a G-box variant (G-boxV;CATGTG) within 94- and 46-bp apart from MybBS, respectively (Supplemental Figure S7, A and B; Schweizer et al., 2013).

**Figure 7:**
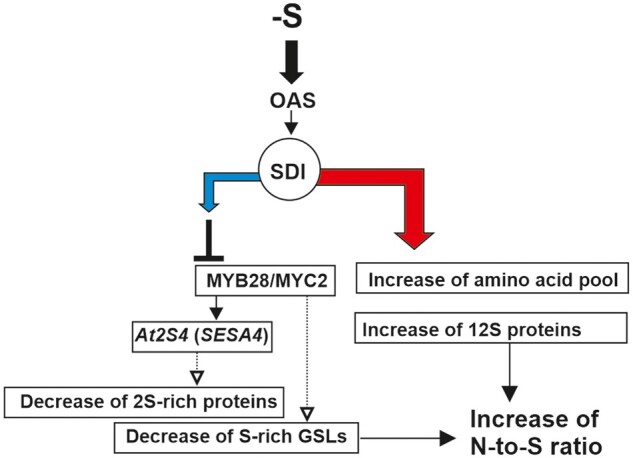
A model represents the functions of SDI in seeds. Under sulfur deficiency (−S), the level of OAS increases, which induces the expression of *SDI1*. SDI1 forms a ternary protein complex with MYB28 and MYC2 and inhibits them through unknown mechanisms. Thereby, the S-rich 2S SSPs, such as At2S4 (SESA4) are reduced and S-poor 12 S SSPs are accumulated. SDI overexpression also leads to accumulation of free amino acid pools which overall enhances the N-to-S ratio. A common phenotype observed under sulfur deficiency in seeds. The blue and red arrows indicate downregulation and upregulation, respectively.

A MYB core cis-element was detected in motif 2 in all the promoter sequences of SESA proteins (Figure 7C). EMSA assay revealed a direct interaction of MYB28 with the labeled probe (*At2S4* promoter), validating the direct regulation of *At2S4* via MYB28 (Figure 6B).

## Discussion

Our data demonstrate that SDI1 protein besides the previously identified downregulation of GSLs in Arabidopsis leaves and roots under −S has a considerable impact on seed metabolome and protein composition (Aarabi et al., 2016). SDI1, which is induced under sulfur-limiting conditions in seeds, not only inhibits GSL accumulation, but also regulates the accumulation of sulfur-rich 2S albumins mainly by suppression of SESA4 and SESA5 although it also moderately suppresses SESA2, SESA3, and At12S3. This observation is in line with the study of Higashi et al, (2006) in which At12S3 and to a greater extent SESA proteins were remarkably reduced in response to sulfur stress (Higashi et al., 2006). Although we were unable to quantify the accumulation of 12S and 2S proteins by MS probably due to saturation of these abundant proteins, SDS–PAGE clearly showed that *SDI1* overexpression leads to the accumulation of 12S, while *sdi1sdi2* dKO leads to accumulation of 2S proteins. Similarly, it is noteworthy that, suppression of 2S albumins, known as S-rich proteins, and accumulation of 12S-globulins, known as S-poor proteins are common phenotypes that occur under sulfur starvation in multiple plant species such as Arabidopsis, and wheat (*Triticum aestivum*; Castle and Randall, 1987; Naito et al., 1994; Hirai et al., 1995; Higashi et al., 2006; Bonnot et al., 2017) and SDI appears to have a fundamental role in triggering this sulfur response phenotype at both transcript and protein levels.

OAS is, additionally, considered a regulator of SSP gene expression (Hirai et al., 2003). The application of OAS to immature soybean cotyledons resulted in a similar pattern of SSP accumulation to that seen under sulfur deficiency (Kim et al., 1999; Hirai et al., 2003), which furthers the notion that SDI is the main determinant in the OAS-dependent downregulation of S-rich proteins in seeds. Furthermore, the upregulation of sulfate assimilation genes in developing seeds of *SDI1ox* lines at 11 and 21 DAF mimics the transcript responses observed under sulfur deficiency in developing wheat grains and Arabidopsis leaves (Nikiforova et al., 2003; Bonnot et al., 2020). Metabolite data also demonstrate that SDI overexpression leads to the upregulation of OAS in seeds, therefore triggering the −S response. On the other hand, knockout lines also demonstrated higher OAS levels. Presumably, the early increased production of S-rich proteins might pose a higher demand on S-rich amino acids, which might mimick a sulfate starvation situation leading to higher OAS synthesis through the perturbation of the regulation of the sulfur assimilation system (Aarabi et al., 2020). The upregulation of free amino acids in seeds of ox lines also could be the result of their lesser incorporation into S-rich proteins, including both S-containing amino acids, Cys, and Met, and N-rich amino acids Asn, and Gln. This phenotype is in line with the previously reported increased amino acid pool in sulfur-starved wheat grains, in which *SDI2* was found to be strongly upregulated during grain filling under S deficiency, and has been proposed as a putative regulator in grain protein accumulation under −S (Bonnot et al., 2020). Bonnot et al. (2020) consider the increased N-to-S ratio in wheat as a regulatory mechanism to adjust the amino acid and protein reserves in grains in response to −S (Bonnot et al., 2017, 2020). Our data reveal that SDI1 and most probably SDI2 have substantial roles in controlling this response in seeds as SDI1 overexpression leads to the upregulation of genes involved in nitrate assimilation and hence accumulation of free amino acids, whereas S-rich proteins and other pools of sulfur such as GSLs are reduced (Figure 7).

We identified that SDI downregulates S-rich proteins in seeds by forming a ternary protein complex with MYB28, an identified regulator of seed proteins in this study, and MYC2, another regulator of seed proteins (Gao et al., 2016), leading to a synergistic downregulation of *2S* genes and thereby, SESA proteins. Therefore, SDI1, MYB28, and MYC2 appear to have additional roles in seed protein accumulation apart from GSL regulation in vegetative tissues. MYB28 directly interacts with MYC2 (Schweizer et al., 2013), and we demonstrated that it functions as a bridging protein between SDI1 and MYC2; however, SDI1 does not interfere with MYB28–MYC2 interaction. Furthermore, it had previously been shown that the SDI1–MYB28 complex formation did not intervene with DNA binding of MYB28; however, it inhibited the MYB28-mediated transactivation of the promoters of the aliphatic GSL biosynthetic genes (Aarabi et al., 2016). Additionally, SDI1 is known to have negative effects on *MYB28* expression (Aarabi et al., 2016), which was also reflected in the transcript data of the seeds in this study. It appears that an antagonistic relationship exists between the expression of *SDI1 and MYB28* over different seed developmental stages (Supplemental Figure S4). Overall, the SDI1 repression mechanism on the MYB28–MYC2 complex needs further investigation. A sterical hindrance of the transcription machinery by the SDI1–MYB28 complex formation had been proposed as a mechanism to block the function of MYB28 as an activator (Aarabi et al., 2016). Alternatively, SDI1 might act as a co-repressor by binding to and activating an unknown repressor of *MYB28 or MYC2*. MYC2 is a multifunctional protein and a master regulator of jasmonate (JA)-mediated signaling involved in the regulation of multiple pathways depending on its interaction partners, thus integrating different environmental signals (Kazan and Manners, 2013). MYC2 is not functional as an activator without an interaction partner (Pireyre and Burow, 2015; Frerigmann, 2016). Several repressors and mediator proteins are known to interact with MYC2 in a big protein complex, such as Jasmonate ZIM-domain (JAZ) proteins, Novel Interactor of JAZ (NINJA), TOPLESS (TPL), and TPR (TPL related), blocking the activation of MYC2 in JA-mediated signaling involved in GSL accumulation (Pireyre and Burow, 2015; Frerigmann, 2016). Whether SDI1 participates as a mediator in such a protein complex to confer its inhibitory effect on the MYB28–MYC2 complex in seed protein accumulation remains to be elucidated.

Given that *sdi1sdi2* dKOs exhibit normal physiological growth and that their central metabolism is not altered, *SDI* loss-of-function presents an approach in fortifying the levels of essential amino acids in SSPs in crop species. Protein sequence comparison of the SSP proteins shows that 2S proteins contain higher levels of sulfur than 12S proteins, as the average of cysteine and methionine content per 100 amino acids in 2S proteins is approximately three to four times higher than that in 12S proteins (Supplemental Table S4). Among the 12S proteins, At12S3 contained higher levels of cysteine and methionine per 100 amino acids compared to the other 12S proteins (Supplemental Table S4). It appears that SDI1 specifically downregulates these S-rich proteins, including SESA2, SESA3, SESA4, SESA5, and At12S3 at a later phase of seed ripening. These proteins also contain higher levels of lysine (Lys) per 100 amino acids compared to other proteins (Supplemental Table S4). Given that Met and Lys are the most limiting essential amino acids in cereals and legumes (Galili and Amir, 2013), *sdi1sdi2* dKO represents a suitable candidate in fortifying these nutritionally essential compounds in seed crops.

We also demonstrated that SDI has dramatic effects on seed secondary metabolites. SDI not only downregulates S-rich GSLs in seeds but also pools of sinapate esters undergo alteration. However, the most abundant sinapate esters in Arabidopsis seed reserves have been reported to be sinapoyl–choline. Our data show that SinM, the other branching metabolite from sinapoyl-Glc undergoes alteration by overexpression of SDI1 at both transcript and metabolite levels. This might be a specific sulfur deficiency response in Arabidopsis seeds which we are reporting here for the first time. *SDI1* overexpression leads to the upregulation of *SNG1*, encoding SMT enzyme to catalyze the conversion of sinapoyl-Glc to SinM (Fraser et al., 2007), leading to strong overaccumulation of sinpoylmalate at the expense of sinapoyl-Glc in dry seeds of the *SDI1ox* lines. Given that sinapate esters are derived from the shikimate/phenylpropanoid pathway, which starts from phenylalanine, a common precursor for indole GSL biosynthesis, further studies need to assess whether the upregulation of SinM in ox lines is an indirect effect of the downregulation of GSLs, which are known as competing pathways with phenylpropanoids (Kim et al., 2015), or is a direct effect of SDI. Sinapoyl–choline and other sinapate esters are known to add antinutritive properties to the seed protein composition of the oilseed crop *B. napus* (oilseed rape; Canola), hampering them to be used for animal feed and human nutrition (Milkowski and Strack, 2010). Therefore, to increase the nutritional value of *B.* *napus* seeds several attempts have been made to generate crops with low sinapate ester content (Milkowski and Strack, 2010). Here, we propose SDI as a candidate to fulfill this aim.

Genome-wide analysis of SDI transgenic lines demonstrates that SDI has moderate effects on global gene expression in seeds, rather, some genes involved in specific metabolic pathways undergo alteration. Except for the genes that are involved in SSP, and SinM synthesis, mentioned above, some genes involved in carbohydrate metabolism such as *UGP1*, and *UGP3* were strongly downregulated in the ox lines. *UGP* genes encode UDP-glucose pyrophosphorylase producing UDP-glucose (Meng et al., 2009), and specifically, *UGP3* is the first committed enzyme for sulfolipid biosynthesis (Okazaki et al., 2009). Furthermore, *UGP3* was found to be the only gene of the sulfolipid pathway, down-regulated in response to short-term sulfur starvation (Okazaki et al., 2009). This observation may indicate a role for SDI in the regulation of yet another pool of sulfur in plants under S deficiency, namely sulpholipids.

The time-course sampling of WT seeds from the onset of seed filling to the desiccation period demonstrated that *SDI1 and SDI2* transcripts peaked at the late maturation phase (18 DAF). On the other hand, time-course SDS–PAGE analysis on sulfur-starved wheat grains demonstrated that sulfur deficiency provokes storage protein synthesis at earlier time-points of seed development compared to the control grains (Castle and Randall, 1987). Thus, it has been proposed that S deficiency modulates the timing of the developmental switch of wheat by shortening the early phase of cell division and activating the seed filling and maturation phases (Castle and Randall, 1987). This partly explains the rationale behind the high expression of *SDI* genes only at the later stage of seed maturation. Hence, a regulatory mechanism should exist in seeds to fine-tune *SDI* expression over seed development. At conditions of high demand for S-rich protein synthesis, e.g. at early to mid-time point of seed maturation under favorable nutritional conditions, *SDI* is repressed through unknown mechanisms, and under conditions of S limitation or when sulfur pools have been used up for S-rich SSPs, e.g at late to post maturation phase, *SDI* gets activated to balance the ratio of S-rich to S-poor proteins and perhaps ending the cellularization period. Furthermore, a common response to −S is an increase of the root-to-shoot ratio, as shoot growth is more reduced than the root growth (Hawkesford et al., 2012; Gruber et al., 2013; Forieri et al., 2017; Aarabi et al., 2020; ), a phenotype that is reflected in SDI1ox line in this study. Growth regulation of Arabidopsis under −S has been revealed to be regulated by glucose–target of rapamycin signaling (Dong et al., 2017). Whether SDI is involved in this regulatory mechanism needs further investigations.

## Materials and methods

### Plant material and growth condition

Arabidopsis lines were used in the WT (Col-0 ecotype) background. Seeds were grown directly on soil and stratified for 1 week at 4°C for vernalization. Plants were then transferred to standard greenhouse conditions (140 μE m^−^^2^ s^−^^1^ light intensity, 40% relative humidity, 24°C) with 16-/8-h light/dark cycles (long-day). Developing seeds at 9 DAF were harvested for the RNA-seq and RT-qPCR analyses. Dry mature seeds were harvested for metabolomics and proteomics studies. To harvest the developing seeds at 11 and 21 DAF plants were grown in a climate chamber with 16 h/8 h of light/dark cycles provided by 120 μE m^−2^s^−1^ light intensity, and a day/night temperature of 20/16°C and relative humidity of 60/75%.

### Generation of overexpression lines

Full-length coding cDNA sequence of *SDI1* was amplified with the primers listed in Supplemental Table S5 and cloned into the pENTR/D-TOPO vector (Invitrogen). Entry clones were then sub-cloned into the Gateway pK7WG2 vector (Karimi et al., 2002; Invitrogen; Supplemental Table S5). Constructs were transformed into *A. tumefaciens* strain GV3101 by Electroporation (modified from Mattanovich et al., 1989) and subsequently into Arabidopsis (Col-0) flower buds by the floral dipping method (Clough and Bent, 1998). Homozygous T3 transgenic plants were selected on a medium containing kanamycin sulfate (50 mg·L^–^^1^). T4 seeds were used for the metabolomics, and proteomics analyses. The generation of the *35S:MYB28*, *35S:MYB29*, and *35S:MYB76* overexpression constructs have been described previously (Sonderby et al., 2007).

### Isolation of homozygous knockout lines and generation of dKO lines

T-DNA knockout lines, SALK_145035 (*sdi1-1*), SALK_099766 (*sdi1-2*), and SALK_091618 (*sdi2-1*), which are in Col-0 background, were identified from the Salk T-DNA lines (Alonso, 2003) by the analysis of the SiGnAL database (http://www.signal.salk.edu/cgi-bin/tdnaexpress). Homozygous lines were obtained via PCR screening on genomic DNA using gene-specific forward and reverse primers followed by T-DNA left border primer and gene-specific forward or reverse primers (Supplemental Table S6). To generate *sdi1sdi2* dKOs, homozygous single knockouts of *sdi1-1* and *sdi1-2* were crossed with the single knockout line of *sdi2-1* and two independent dKO lines (dKO1 for *sdi1-1sdi2-1* and dKO2 for *sdi1-2sdi2-1*) were established and selected for further analysis. The generation of the T-DNA insertion mutants in At5g61420 (line SALK_136312, *myb28-1*), At5g07690 (SM.34316 = *myb29-2*), and the dKO *myb28-1myb29-2* have been described previously (Sonderby et al., 2007).

### Isolation of developing seed RNA and cDNA synthesis

To harvest the green developing Arabidopsis seeds for RNA extraction, flower buds were tagged with tape after the onset of flowering, and seeds were dissected from siliques in different days, corresponding to approximately 9, 11, 13, 18, and 21 DAF. Total RNA was extracted using the Spectrum Plant Total RNA Kit (SIGMA). Residual DNA was removed by On-Column DNase Digestion Set (SIGMA). Two microgram of total RNA treated with RNase-Free DNase (Qiagen) was utilized as a template for the first-strand cDNA synthesis using the Maxima cDNA synthesis kit (Thermo Scientific) according to the manufacturer’s instructions.

### RT-qPCR

RT-qPCR was performed using 0.5 μL of the generated cDNA (∼50 ng µL^-^^1^), 2 μL of each gene-specific primer (0.5 μM), and 2.5 µL of the 2X SYBR Select Master Mix (Applied Biosystems). PCR was run with an ABI PRISM 7900HT Fast Real-Time PCR System (Applied Biosystems). PCR thermal-cycling condition was performed according to the SYBR Green’s manufacturer’s instructions. SDS 2.2.1 software (Applied Biosystems) was used for data analysis. Relative expression values are presented as 2^−^^ΔCT^; ΔCT = CT (gene of interest) –CT (UBQ10 or AT3g12210). The primer sequences used are listed in Supplemental Table S7.

### RNA-seq analysis and data analysis

Nine RNA libraries including polyA enrichment were generated from total RNA extracts and sequenced on Illumina HiSeq3000 in 2 × 150 bp (paired-end read) in Max Planck-Genome-centre Cologne (MP-GC). The sequencing data were uploaded to the Galaxy web platform (Afgan et al., 2018), and the GREEN HUB Galaxy server belonged to the TRR175 The Green Hub consortium, was used for data analysis. The obtained reads were mapped to the Arabidopsis genome using STAR (Dobin et al., 2012), and the number of reads per annotated gene was counted using featureCounts (Liao et al., 2013). Differential gene expression analysis has been performed using DESeq2 against the corresponding WT at 9 DAF (Love et al., 2014). The depicted MA plots in Supplemental Figure S2 and the PCA plot in Figure 2A were acquired as outputs of DESeq2 analysis. g:Profiler was used to find the biological processes and molecular functions that are overenriched in the differential analysis results (Raudvere et al., 2019).

### Metabolite analysis

Methyl-tertiary-butyl-ether (MTBE) extraction method was employed for measurements of ions, primary and secondary metabolites, and lipids as described in Salem et al. (2016, 2017). Aliquots (10 mg, each) of the frozen-homogenized dry or developing seeds were fully suspended in 1 mL of pre-chilled (−20°C) methanol: MTBE (1:3 [v:v]) mixture and incubated for 10 min in an orbital shaker at 4°C. A mixture of 500-μL water: methanol (1:3 [v:v]) was added and mixed well with the samples. After centrifugation for 10 min (13,000*g*), the upper organic phase (500 μL) was concentrated for lipid measurements and finally re-suspended in 600 μL of acetonitrile (ACN): isopropanol (7:3 [v:v]). A volume of 2 µL per sample was injected in the UPLC/ESI-MS system (Waters Acquity UPLC system coupled to an Exactive [Thermo-Fisher] high-resolution mass spectrometer; Salem et al., 2016, 2017). Secondary metabolites were measured as described in Tohge et al. (2016) and Tohge and Fernie (2010). Aliquots (150 μL) of lower polar phases were dried in a speed-vac concentrator, re-suspended in 100 μL of 80% (v/v) methanol containing isovitexin as internal standard. For the analysis, 5 μL were injected for LC/ESI–MS analysis using linear ion trap ESI–MS system Finnigan Ltq (Thermo Finnigan) connected to a Surveyor HPLC System (Thermo Fisher; Tohge and Fernie, 2010). Chromatograms were recorded and processed with Xcalibur (Version 2.10, Thermo-Fisher), ToxID (Version 2.1.1, Thermo-Fisher), or the Refiner MS software (Version 6.0, Gene-Data, Basel, Switzerland; Hummel et al., 2011). Peak areas were normalized based on the fresh weight of the sample and the internal standard. Primary metabolites were measured according to Lisec et al. (2006) and mass tags identified according to the Golm Metabolome Database (Hummel et al., 2011).

### Seed protein extraction and SDS–PAGE

Proteins were extracted from mature Arabidopsis seeds as described by Naito et al. (1988). Five mg of dry seeds were homogenized in 100 μL of extraction buffer (100-mM Tris–HCl, pH 8, 0.5% (w/v) SDS, and 10% (w/v) glycerol) and boiled for 3 min at 99°C. Samples were centrifuged at 15,000*g* for 5 min at 4°C. The supernatant was taken as the extracted protein. The protein concentration was determined by Bradford assay (Bradford, 1976) using Bovine serum albumin (BSA) as a standard. Ten micrograms of protein were separated by SDS–PAGE in 10% (w/v) polyacrylamide. SeeBlue Plus2 Pre-stained Protein Standard (Life technologies) was used as a molecular weight standard. Proteins were visualized using Coomassie Brilliant staining (Neuhoff et al., 1985).

### EMSA

Purification of MYB28 protein was performed as follows. Gateway recombinant construct of pDEST 24 (Invitrogen) containing C-terminal GST tagged AtMYB28 (Supplemental Table S5) was introduced to *Escherichia* *coli* rosetta (DE3) cells. The positive transformant was cultured in LB media included ampicillin (100 µg·mL^−^^1^) and chloramphenicol (50 µg·mL^−^^1^) overnight (37°C). A total of 150 µL of the overnight pre-culture was re-cultured to 3-mL fresh LB media in the aforementioned condition for 2 h. 1 mM IPTG was added to the culture for inducing AtMYB28 expression and incubate at 30°C for 5–6 h. The induced culture was collected and pelleted by microcentrifugation. The cell pellets were resuspended in 150-µL extraction buffer comprising 20-mM sodium phosphate buffer (pH 7.4), 1-mM phenylmethylsulfonyl fluoride, 1-mM ethylenediaminetetraacetic acid, 0.5-M sodium chloride, protein inhibitor. Cell disruption was executed via lysozyme and ultrasonication, respectively. Extracts of crude, supernatant, and pellet were collected independently. The fraction of supernatants was utilized to do EMSA. Furthermore, the supernatants of *E. coli* transformed with the cloning entry vector were used as a negative binding control. This was used to ensure no interactions between MYB28-conjugated GST and a DNA probe. The presence of full-length MYB28 protein was validated by a western blot signal, via the 800 nm channel of the Odyssey 9120 (LI-COR), at the predicted molecular weight with a fluorochrome-conjugated secondary antibody (GST-tag Monoclonal antibody, Novagen) against GST, which is attached to a primary antibody (Anti-DCX antibody produced in goat, Sigma; Supplemental Figure S8). Double-stranded probes were generated by annealing 10-µM sense and antisense oligonucleotides, labeled and unlabeled, at 95°C for 5 min in TE buffer, and decrease the temperature to 4°C by −1°C/cycle for 20 s, via the T100TM thermocycler (Bio-rad). The labeled probes then were diluted by 1:200, as compared to unlabeled probes. The EMSA reaction was performed by the Odyssey Infrared EMSA kit (LI-COR). The binding interactions between a candidate TF and a small promoter region were detected using a fluorescently labeled (5′ DY-682) DNA probe, produced by Eurofins Genomics. Electrophoresis was executed with a 6% DNA retardation gel (Invitrogen), and run in the TBE buffer at 4°C. The competitors and mutated competitors, which are oligo-nucleotides without a probe, were used to confirm those bindings. Probe sequences are listed in Supplemental Table S8.

### Liquid chromatography and MS analysis of seed proteins

The digested peptides were acidified to pH < 3.0 with 10% (v/v) trifluoroacetic acid (TFA). The peptide mixture was purified and desalted on C18 SEP-Pak columns (Tecknokroma), which were attached to a QIAvac 24 Plus (QIAGEN) vacuum manifold. The columns were equilibrated with 1-mL 100% (v/v) methanol, once with 1-mL 80% (v/v) ACN and twice with 1 mL of 0.1% (v/v) TFA. The peptides were applied to the C18 column and allowed to pass through slowly. The column was washed twice with 1 mL of 0.1% (v/v) TFA. Peptides were eluted with 800-µL 60% ACN (v/v), 0.1% (v/v) TFA, dried in the speed vacuum concentrator and stored at −80° prior to MS analysis. Peptides were resuspended in 30 μL of resuspension buffer (5% [v/v] ACN, 2% [v/v] TFA). Measurements were performed on a Q Exactive HF coupled to an Easy nLC1000 HPLC (Thermo Scientific). Eight microliters of the samples were loaded onto an Acclaim PepMap RSLC reversed-phase column (75-μm inner diameter, 15-cm length, 2-µm bead size [Thermo Scientific]) at a flow rate of 0.8-μL min^−^^1^ in a buffer consisting of 3% (v/v) ACN, 0.5% (v/v) acetic acid. Peptide elution was facilitated by increasing the ACN gradient from 3% to 30% (v/v) over 100 min, from 30% to 40% for the next 10 min and from 40% to 80% for the last 5 min at a flow rate of 0.3 μL·min^−^^1^. The column was then washed with 80% (v/v) ACN for 5 min, at a flow rate of 0.5 μL·min^−^^1^. Peptide ions were detected in a full scan from the mass-to-charge ratio of 150 to 1,600 at a resolution of 60,000. Tandem mass spectrometry (MS/MS) scans were performed for the 15 peptides with the highest MS signal at a resolution of 15,000 (AGC target 2e5, isolation width mass-to-charge ratio 3 *m/z*, relative collision energy 30%). Peptides for which MS/MS spectra had been recorded were excluded from further MS/MS scans for 20 s. Quantitative analysis of MS/MS measurements was performed with the Progenesis liquid chromatography/mass spectrometry (LC/MS) software (Nonlinear Dynamics). The selection of a reference run and, alignment and peak picking was performed automatically. The spectra for each MS1 signal peak were exported to Mascot (Matrix Science). Mascot search parameters were set as follows: Arabidopsis TAIR10 genome annotation (Garcia-Hernandez et al., 2002), requirement for tryptic ends, one missed cleavage allowed, fixed modification: carbamidomethylation (cysteine), variable modification: oxidation (methionine), peptide mass tolerance = ±10 ppm, MS/MS tolerance = ±0.8 Da, allowed peptide charges of +2 and +3. Spectra were also searched against a decoy database of the Arabidopsis proteome and results were filtered to ensure an FDR <1% on the protein level. Additionally, peptide identifications with a Mascot score <40 were excluded. Mascot results were imported into Progenesis QI, quantitative peak area information extracted and the results exported for further analysis.

### Y2H and Y3H analyses

Y2H and Y3H were performed using the Matchmaker Gold Yeast Two-Hybrid System according to the manufacturer’s instructions (Clontech). For the Y2H, SDI1-BD clone was used as the bait, described previously (Aarabi et al., 2016), and mated with prey construct, MYC2-AD (Supplemental Table S5). For the Y3H Two combinations of constructs were generated in the pBridge vector (Clontech; Supplemental Table S5). SDI1BD-MYB28 expressed SDI1 fused to the DNA-BD (Gal4 DNA-binding domain) as well as MYB28 expressing as the bridging protein, without any attachment to the binding domain or activation domain. MYB28BD-SDI expressed MYB28 fused to the DNA-BD domain and SDI1 as the bridging protein. These two constructs were co-transformed with MYC2AD (Supplemental Table S5) as described previously (Aarabi et al., 2016). Positively transformed colonies were selected on the double, triple, and quadruple dropout plates as −Leu/−Met (DDO), −Leu/−Trp/−Met (TDO), and −His/−Leu/−Met/−Trp (QDO), respectively, either with or without X-α-Gal (X) and Aureobasidin.

### Accession numbers

The list of genes examined in this study and the corresponding accession numbers is summarized in Supplemental Table S7.

## Supplemental data

The following materials are available in the online version of this article.

**Supplemental Figure S1.** Transcript levels of *SDI1* (black line) and *SDI2* (orange line) in Arabidopsis seed regions and sub-regions throughout seed development.

**Supplemental Figure S2.** Global view of the relationship between the expression changes of *SDI* transgenic lines and the WTs at 9 DAF.

**Supplemental Figure S3.** Scatter plot showing the fold change (log 2) of RNA-Seq compared to the fold change (log 2) of RT-qPCR quantified genes.

**Supplemental Figure S4.** Expression pattern of *SDI1* in comparison to the TFs regulating SSPs.

**Supplemental Figure S5.** Sulfate, nitrate, total triacylglycerol, and protein contents of dry seeds of the SDI transgenic lines.

**Supplemental Figure S6.** Quantification of the SDS–PAGE protein bands.

**Supplemental Figure S7.** Motif enrichment analysis in promoter sequences of seed SESA proteins.

**Supplemental Figure S8.** A western blot represents the enrichment of MYB28 protein.

**Supplemental Table S1.** Differential gene expression of the genes encoding SSPs, and the corresponding TFs responsible for encoding SSP genes in developing seeds of the SDI transgenic lines quantified by RNA-seq.

**Supplemental Table S2.** Differential transcript levels (fold changes) of some selected genes in seeds of SDI transgenic lines at 9 DAF, assayed by q-RT PCR. The assay performed using three independent biological replicates and two technical replicates. Significantly different DEGs versus WT (at *P* < 0.05, detected by Student’s t test) are highlighted in green. The heatmap threshold were set between 0 and 10 as the minimum indicated in blue, and 10 as the maximum fold changes, indicated in red.

**Supplemental Table S3.** Differential transcript levels (fold changes) of some selected genes in seeds of SDI transgenic lines at 11 and 21 DAF, assayed by q-RT PCR. The assay performed using two independent biological replicates and two technical replicates. Significantly different DEGs versus WT (at *P* < 0.05, detected by Student’s *t* test) are highlighted in green. The heatmap threshold were set between 0 and 10 as the minimum indicated in blue, and 10 as the maximum fold changes, indicated in red.

**Supplemental Table S4.** Comparison of protein sequences of the 12S and 2S SSPs.

**Supplemental Table S5.** Oligonucleotides used for vector construction. Recognition sites for restriction enzymes are underlined.

**Supplemental Table S6.** Oligonucleotides used for isolation of the T-DNA insertion lines.

**Supplemental Table S7.** Oligonucleotides used for qRT-PCR analysis.

**Supplemental Table S8.** Oligonucleotides used for EMSA.

**Supplemental Data Set S1.** SDI-regulated genes at 9 DAF (cotyledon stage).

**Supplemental Data Set S2.** Raw metabolite data of dry seeds of the SDI transgenic lines.

## Supplementary Material

kiab386_Supplementary_Data

## Notes

F.A. and R.H. designed the research. F.A. wrote the manuscript supported by the co-authors, generated the transgenic lines and the constructs, performed metabolite extraction and sample preparation for metabolite measurements, RNA extraction, RT-qPCR, Y2H screening, and analyzed the data. F.A., A.R.F., N.O., and Z.N. performed RNA-seq analysis. A.R. performed EMSA, and western blot. R.B. performed the SDS–PAGE analysis. M.G. and A.G. performed the MS analysis. S.A., T.T., M.W., R.H., and A.R.F. measured metabolite levels. Y.Z. and A.R.F. performed Y3H screening. M.A.S. and P.G. performed lipidomics. F.B. performed seed RNA and protein extractions. A.R.F. and R.B. implemented ideas. A.R.F., R.H., Z.N., and P.G. edited the manuscript.

The author responsible for distribution of materials integral to the findings presented in this article in accordance with the policy described in the Instructions for Authors (https://academic.oup.com/plphys/pages/general-instructions) is: Fayezeh Aarabi (Arabi@mpimp-golm.mpg.de).
